# Monitoring Glucose Concentrations in Children with Epilepsy on a Ketogenic Diet

**DOI:** 10.3390/healthcare10020245

**Published:** 2022-01-27

**Authors:** Katharina Schiller, Tamir Avigdor, Aline Kortas, Mirjam Kunz, Gabriele Unterholzner, Martin Klingelhöfer, Markus Rauchenzauner

**Affiliations:** 1 Department of Pediatrics, Medical University Innsbruck, 6020 Innsbruck, Austria; katharina.schiller@kliniken-oal-kf.de; 2 Department of Pediatrics and Neonatology, Hospital Kaufbeuren, 87600 Kaufbeuren, Germany; aline.kortas@kliniken-oal-kf.de (A.K.); mirjam.kunz@kliniken-oal-kf.de (M.K.); gabriele.unterholzner@kliniken-oal-kf.de (G.U.); martink_94928@yahoo.com (M.K.); 3 Department of Neurology, Montreal Neurological Hospital and Institute, Montreal, QC H3A2B4, Canada; tamir.avigdor@mail.mcgill.ca

**Keywords:** epilepsy, flash glucose monitoring, children, Ketogenic diet, pulsatile corticoid therapy, hypoglycemia, hyperglycemia

## Abstract

Ketogenic diet (KD) and pulsatile dexamethasone therapy (PDT) are commonly used in the treatment of children with drug resistant epilepsy. Potential side effects of the KD are hypoglycemia, whereas PDT might lead to hyperglycemia. One practical option to measure glucose concentrations regularly is the flash glucose monitoring system (FGM). In this single-center study in Germany, two pediatric patients with epilepsy (age: 6.0 and 6.8 years) received FGM from the beginning of the KD over six months, in the year 2020, and one patient (9.8 years) was observed for one month on PDT and switched to the KD thereafter. Glucose concentrations were measured by using an FGM system and capillary blood measurement. Seizure frequency, changes in cognition, motor performance, social behavior, and sleep quality were evaluated. The mean hypoglycemia rate per day (65 mg/dL and lower) declined significantly in patient 1 and 2 after three months. Patient 3 showed in total seven hyperglycemic events during PDT. Patient 1 became seizure free. Improvement of attention and memory performance were reported. FGM during the KD as a treatment for drug resistant epilepsies in childhood is a practical option to explore and to avoid hypoglycemia during the KD and hyperglycemia during PDT.

## 1. Introduction

Epilepsy is one of the most common neurological diseases found in childhood. Up to one third of the patients with epilepsy treated show pharmaco-resistance [1]. One frequently used and effective treatment for drug resistant epilepsies in childhood is the Ketogenic diet (KD), comprising a high amount of fat and low percentage of carbohydrates [2]. In a review by Rezaei et al. evaluating the success of the KD, the majority of the children and adolescents showed a 50% decrease in seizure frequency up to seizure freedom, during the first six months after beginning the KD [3]. The KD was also shown to be effective and well-tolerated in infants with intractable epilepsy [4]. A very low-calorie KD was used as a therapeutic approach for many diseases, such as epilepsy or obesity [3,5,6]. There are different types of the KD and the classic KD used for therapeutic diet in drug resistant epilepsy consists of a main proportion of fat, which is commonly a 4:1 ratio, and for children, a 3:1 ratio of fat to carbohydrate, plus protein [7]. Metabolic changes related to the anticonvulsant properties of the KD include ketosis [8].

Despite the positive results on seizure reduction, one potentially harmful consequence of the KD is the incidence of either acute and severe or chronic and non-severe hypoglycemia [9,10,11]. The negative impact of hypoglycemia on the brain and cognitive development has been demonstrated frequently in children with type 1 diabetes mellitus [12,13]. Particularly during childhood and additionally in combination with seizures, severe and chronic hypoglycemia was found to be related to a decline in cognition and learning skills [13]. Despite the fact that hypoglycemia in the pediatric seizure patient is rare [14] and childhood epilepsy is no more frequent in children with type 1 diabetes [15], regular glucose measurement is recommended to prevent seizures and status epilepticus related to hypoglycemia [16,17].

A practical method to continuously observe glucose concentration is the interstitial glucose measurement using a flash glucose monitoring system (FGM), such as the FreeStyle libre^®^ (Abbott Diabetes Care, Wiesbaden, Germany). In this system, a wired enzyme sensor patch is placed in the musculus triceps brachii, and glucose measurements are shown by swiping a scanner over the sensor. The advantages of the FGM are the precision and stability of the measurements, as well as usage for 14 days without calibration [18]. The effectiveness of using a FGM during the KD was previously shown in a patient with Dravet Syndrome [19], and with continuous glucose measurement in a patient with intractable epilepsy [20], highlighting that the hypoglycemia rate is increased, particularly at the beginning of the KD.

Another effective treatment of drug resistant epilepsy in children is intravenous corticoid therapy. Several intractable epilepsy syndromes of childhood, such as continuous spike-and-wave during sleep, West Syndrome, and electrical status epilepticus during slow-wave sleep (ESES), when treated with pulsatile dexamethasone therapy (PDT) showed a significant decrease in seizure frequency or improvement of the electroencephalogram [21,22]. Concomitant with PDT, weight gain, as well as hyperglycemia were observed as side effects [23,24]. Chronic hyperglycemia might also have a detrimental effect on the brain, as smaller white and larger grey matter volume was associated with hyperglycemia in adolescent patients with T1DM [25] and led to reduced memory and learning capacity [26].

To date, data concerning continuous glucose measurement in patients with epilepsy on the KD or PDT to quantify hypo- and hyperglycemia rates are lacking. Therefore, the goal of this case series of three pediatric patients with drug resistant epilepsy on the KD or PDT, is to provide an insight into the control of glucose and ketone concentrations with a FGM system.

## 2. Materials and Methods

The study was approved by the Ethics Committee of the Ostallgäu-Kaufbeuren hospital group, Germany. Patients and their parents gave consent to participation. Patients were recruited from the children’s unit of Kaufbeuren Hospital.

### 2.1. Observational Period

For patient 1 and 2, the observational period began at the initiation of the KD and lasted for six months. Patient 3 received PDT for one month and was subsequently switched to a three-month period of the KD.

### 2.2. Glucose and Ketone Measurement

Glucose and ketone concentrations were controlled using a flash glucose monitoring system (FreeStyle libre^®^). Glucose was determined by using the implanted glucose sensor, FreeStyle libre^®^. For additional calibration, glucose was determined several times per day by capillary blood glucose measurement. Parents reported the capillary blood glucose measurement in a diary with additional notes about clinically related symptoms such as nausea or fatigue. Hypoglycemia was defined according to the International Society for Pediatric and Adolescent Diabetes (ISDAP) Clinical Practice Consensus Guidelines 2014, as a serum glucose level of 65 mg/dL or lower [27]. Hyperglycemia was defined as a serum glucose level of 140 mg/dL or higher [28]. The therapeutic range of the ketone concentrations was set between 2 and 6 mmol/L.

### 2.3. Qualitative Measurement

At the end of the observational period, a questionnaire was filled out by the parents. For patients 1 and 2, the questionnaire was filled out six months after initiating the KD. For patient 3, the questionnaire was filled out three months after the initiation of the KD. The questionnaire was developed in-house and consisted of one item concerning the number and semiology of seizures during the therapy, and 10 Likert-scaled items about the development of cognition (concentration, language, memory), motor skills, social behavior, emotional state, and sleep quality (for example: “How do you rate the sleep quality of your child?” ranging from 1 = extremely bad to 10 = extremely good), each measured before, during, and after the treatment.

### 2.4. Patients—Three Cases

#### 2.4.1. Patient 1

The girl was born by caesarean section. At the age of 5.4 years, generalized seizures occurred. In the electroencephalogram (EEG), interictal epileptiform discharges (IEDs) over both hemispheres were registered. During the following years, an increasing number of seizures occurred including one grand mal seizure with status epilepticus. Family history was negative, cognitive performance and attention was declining, as reported by the mother. MRI of the brain showed no abnormalities. Clinical, biochemical, metabolic, and cardiologic (electrocardiogram (ECG), 24 h ECG, blood pressure measurement and echocardiography), as well as genetic evaluation, revealed no abnormal findings.

After initial treatment with levetiracetam, she was switched to valproate due to aggressive behavior and an increasing frequency of atonic seizures. Consecutively, rufinamide and clobazam were added in combination.

KD—Observational period:

At age 6.0 years, the girl was diagnosed with ESES. A 2.5:1 KD consisting of 2.5 times the amount of fat as carbohydrates was initiated, alongside a treatment with valproate and clobazam. For example, this equates to 2.5 g of fat per 1 g of carbohydrates. Eleven weeks after the beginning of the KD, lamotrigine administration was started and clobazam was withdrawn.

#### 2.4.2. Patient 2

The boy was born by vaginal delivery. Starting at the age of 3.7 years, he showed focal myoclonia with pathological EEG (grade III). MRI of the brain showed no abnormalities. Clinical, ophthalmologic, biochemical, metabolic, and genetic evaluation revealed no abnormal findings. First, he was treated with levetiracetam, clobazam, and zonisamid. Due to continuing seizures, a cortisone therapy was initiated at age 4.8 years, with no significant decrease in interictal activity. During the clinical evaluation of the cortisone therapy, cardiologic evaluation revealed a pulmonary valve insufficiency and a tricuspid insufficiency.

Although the boy was treated consecutively with topiramate, potassium bromid, and perampanel, myoclonic seizures increased in frequency and severity. The patient showed up to 100 myoclonia per day with severe falls. Additionally, ADHD was diagnosed, and amphetamine was administered.

KD—Observational period:

At age 6.8 years, the KD was started in conjunction with a parallel treatment with perampanel, clobazame, and zonisamid. After three months, the 3:1 KD consisting of three times the amount of fat as carbohydrates was switched to a 2.5:1 KD, due to weight gain and dry skin.

#### 2.4.3. Patient 3

The girl was born by vaginal delivery. Postnatally, microcephaly was registered, and the development of language and fine motor skills were delayed. At the age of 7.4 years, ADHD was diagnosed and, therefore, the girl was treated with methylphenidate, which was later switched to guanfacine and then later to risperidone. In the EEG, focal and generalized IEDs, as well as generalized slowing were found, and the girl was diagnosed with ESES. MRI of the brain showed no abnormalities. Biochemical and metabolism evaluation revealed no abnormal findings. Genetic analyses showed a mutation of FBXO11, which can be associated with intellectual disability and behavioral anomalies [29].

Previously, the girl was treated with levetiracetam, valproate, sultiam, and lamotrigine, which led to behavioral problems.

PDT and KD—Observational period:

At the age of 9.8 years, PDT was started with a dose of 20 mg/m^2^ of body surface for three days each week at three-week intervals, administered in 10 cycles. The observational period was 30 days (including two cycles). The PDT effectuated neither significant clinical nor EEG improvement. Therefore, a 3:1 KD was subsequently initiated for three months.

### 2.5. Statistics

Hypoglycemia rate was calculated as the percentage of hypoglycemic measurements of the total number of glucose measurements per day registered by the FGM. Comparisons between hypoglycemia rates were performed using the non-parametric Welsh’s *t*-test. Correlations between glucose and ketone concentrations were calculated using the Spearman correlation coefficient with Holmes multiple comparison correction. All analyses were performed two-tailed with *p*-values ≤ 0.05 indicating statistical significance. Statistical analyses were performed using the Statistical Package for Social Sciences for Windows (SPSS Inc., Chicago, IL, USA, version 27) and R language script.

## 3. Results

### 3.1. Patient 1

#### 3.1.1. Quantitative Measurement

Glucose concentrations

Patient 1 had a total amount of 1979 glucose concentration measurements (Mean ± SD: 74.8 mg/dL ± 12.5 mg/dL; min: 34 mg/dL, max: 134 mg/dL) registered over the observational period of six months. Thereof, 1064 measurements of glucose concentration (Mean ± SD: 73.5 mg/dL ± 12.2 mg/dL; min: 34 mg/dL, max: 125 mg/dL) were registered during the first three months and 915 measurements (Mean ± SD: 76.4 mg/dL ± 12.6 mg/dL; min: 43 mg/dL, max: 134 mg/dL) were registered during the following three months (months 4–6 since the beginning of the KD).

Overall, 473 events of moderate hypoglycemia with a minimum of 34 mg/dL, not related to day or night, were recorded (Mean ± SD: 59.8 mg/dL ± 4.5 mg/dL). During the first three months, 287 events (Mean ± SD: 59.7 mg/dL ± 4.6 mg/dL, min: 34 mg/dL), and during the second three months, 186 events (Mean ± SD: 60.0 mg/dL ± 4.2 mg/dL, min: 43 mg/dL) of moderate hypoglycemia were measured. Moreover, 54 events of significant hypoglycemia (serum glucose 54 mg/dL or lower) occurred throughout the six month observational period, where 35 were registered during the first three months, and 19 were registered during the second three months.

**Figure 1:**
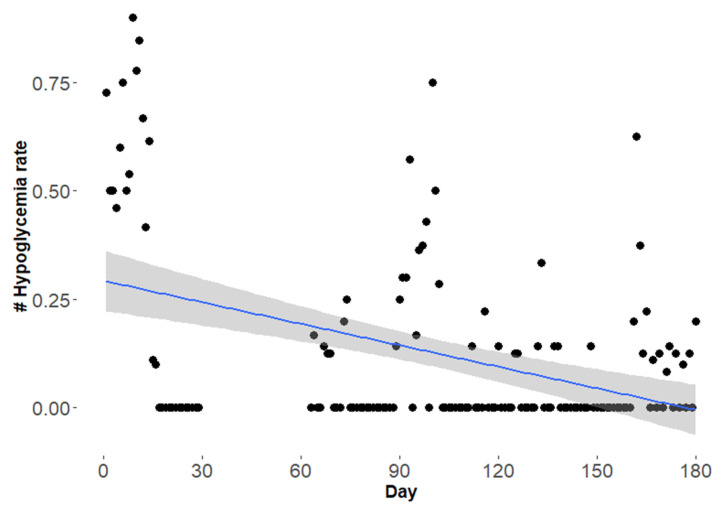
Hypoglycemia rate (glucose 65 mg/dL and lower) per day in patient 1 over the observational period of six months of KD. Each point represents the hypoglycemia rate per day and the regression line (blue) is presented with 95% confidence interval (grey shading).

The hypoglycemia rate (65 mg/dL and lower) per day was calculated for the observational period. The difference between the hypoglycemia rate in the first three months (Mean ± SD: 0.18 ± 0.27) and the last three months (Mean ± SD: 0.09 ± 0.15) was statistically significant (*p* = 0.028) (Figure 1).

Ketone concentrations

In total, 1066 ketone concentrations were registered (Mean ± SD: 3.7 mmol/L ± 1.0 mmol/L, min: 1.1 mmol/L, max: 6.8 mmol/L) over the observational period of six months. During the first three months, 529 ketone concentrations (Mean ± SD: 3.5 mmol/L ± 1.1 mmol/L, min: 1.1 mmol/L, max: 6.6 mmol/L), and during the second three months, 537 ketone concentrations (Mean ± SD: 3.9 mmol/L ± 1.0 mmol/L, min: 1.5 mmol/L, max: 6.8 mmol/L) were measured. The correlation between ketones and glucose concentration was statistically significant (r = −0.23, *p* = 0.004).

#### 3.1.2. Qualitative Measurement

In the first two months after the initiation of the KD, the patient became seizure free. Subsequently, the patient started stumbling with daily falls alongside weekly grand mal seizures once per week, with a minimal duration of 30 s. These events were not related to hypoglycemic events or ketone concentrations beyond the therapeutic range. Eleven months after the beginning of the KD, lamotrigine was added and clobazam was reduced. Since then, the patient has been seizure free until the time of writing.

The mother reported an improvement of cognition (concentration and memory), motor performance, emotional state, and sleep quality. In the questionnaire, concerning language and social behavior, no differences were observed.

### 3.2. Patient 2

#### 3.2.1. Quantitative Measurement

Glucose concentrations

Patient 2 had a total amount of 1336 glucose concentrations (Mean ± SD: 78.3 mg/dL ± 15.9 mg/dL; min: 41 mg/dL; max: 165) registered during the observational period of six months. Thereof, 676 measurements of glucose concentration (Mean ± SD: 76.4 mg/dL ± 15.3 mg/dL; min: 41 mg/dL; max: 134 mg/dL) were registered during the first three months and 660 measurements (Mean ± SD: 80.4 mg/dL ± 16.3 mg/dL; min: 43 mg/dL, max: 134 mg/dL) were registered during the following three months (months 4–6 since the beginning of the KD).

Overall, 319 events of moderate hypoglycemia with a minimum of 41 mg/dL, irrespective of day or night, were recorded (Mean ± SD: 59.3 mg/dL ± 4.9 mg/dL). During the first three months, 188 events (Mean ± SD: 59.1 mg/dL ± 4.8 mg/dL; min: 41 mg/dL), and during the second three months, 131 events (Mean ± SD: 59.5 mg/dL ± 4.7 mg/dL; min: 43 mg/dL) of moderate hypoglycemia were measured. Moreover, 48 events of significant hypoglycemia (serum glucose level 54 mg/dL or lower) were recorded over the six month observational period, of which 29 were registered during the first three months and 19 were registered throughout the following three months.

**Figure 2:**
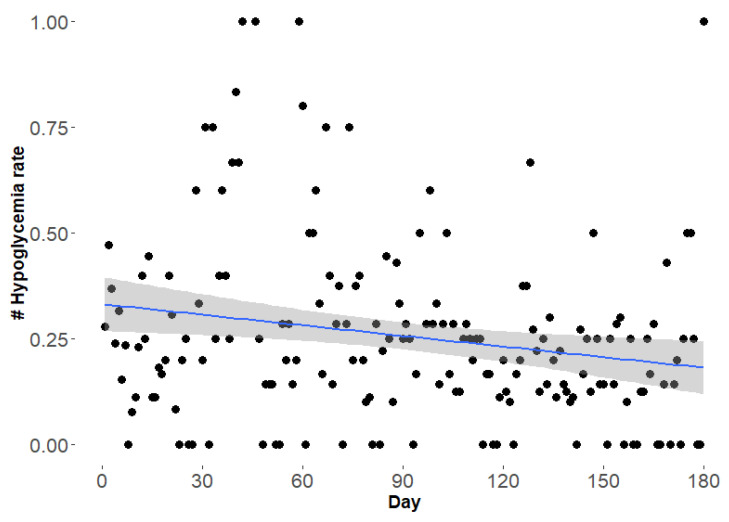
Hypoglycemia rate (glucose 65 mg/dL and lower) per day in patient 2 over the observational period of six months of KD. Each point represents the hypoglycemia rate per day and the regression line (blue) is presented with 95% confidence interval (grey shading).

The hypoglycemia rate per day was calculated for the observational period. The difference between hypoglycemia rate in the first three months (Mean ± SD: 0.30 ± 0.24) and the last three months (Mean ± SD: 0.20 ± 0.16) was statistically significant (*p* = 0.002) (Figure 2).

Ketone concentrations

In total, 906 ketone concentrations were registered (Mean ± SD: 4.7 mmol/L ± 1.1 mmol/L, min: 1.1 mmol/L, max: 9.4 mmol/L) during the observational period of six months. During the first three months, 396 ketone concentrations (Mean ± SD: 4.7 mmol/L ± 1.0 mmol/L; min: 2.0 mmol/L, max: 7.0 mmol/L), and during the second three months, 510 ketone concentrations (Mean ± SD: 4.7 mmol/L ± 1.2 mmol/L; min: 1.4 mmol/L, max: 9.4 mmol/L) were measured. The correlation between ketones and glucose concentration was statistically significant (r = −0.19, *p* = 0.01).

#### 3.2.2. Qualitative Measurement

The number of myoclonic seizures per day was remarkably reduced after starting the KD. The frequency of more than 100 myoclonia per day decreased to 10, including some seizure-free days. Over the observational period, 5–10 generalized seizures with a duration of 4–8 s, not related to hypoglycemic episodes or ketone concentrations, were observed.

The mother reported improved cognition (concentration and memory), motor performance, social behavior, emotional stability, and sleep quality. Language improvement was only marginal.

### 3.3. Patient 3

#### 3.3.1. Quantitative Measurement

Phase 1—PDT

Patient 3 had a total amount of 96 glucose concentrations measured in capillary blood and registered over the observational period of one month.

**Figure 3:**
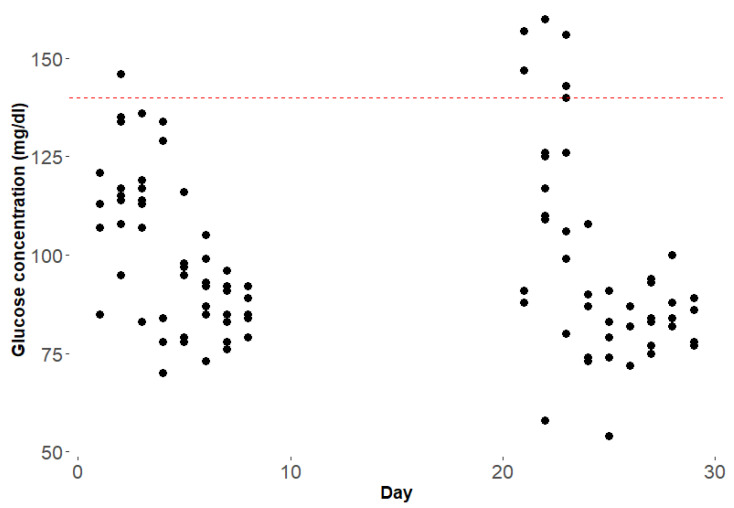
Glucose concentrations of patient 3 during PDT (red line marking hyperglycemia serum glucose 140 mg/dL and higher). Each point represents the glucose concentration measurement.

Overall, seven events of hyperglycemia with a maximum of 160 mg/dL, and two events of hypoglycemia with a minimum of 54 mg/dL, not related to day or night, were recorded (Mean ± SD: 98.9 mg/dL ± 22.7 mg/dL) (Figure 3). Moreover, one event of significant hypoglycemia (serum glucose level 54 mg/dL or lower) occurred during the one-month observational period.

Phase 2—KD

In the phase of the KD, a total measurement of 847 glucose concentrations (Mean ± SD: 85.3 mg/dL ± 11.5 mg/dL; min: 44 mg/dL; max: 149 mg/dL) were registered over the observational period of three months. Overall, 48 events of moderate hypoglycemia with a minimum of 44 mg/dL, not related to day or night, were recorded (Mean ± SD: 60.9 mg/dL ± 4.5 mg/dL). Moreover, five events of significant hypoglycemia (serum glucose level 54 mg/dL or lower) were registered throughout the observational period of three months. The mean hypoglycemia rate per day was 0.2 (SD 0.26). A total of 241 ketone concentrations were registered (Mean ± SD: 3.1 mmol/L ± 1.1 mmol/L; min: 0.8 mmol/L, max: 5.9 mmol/L) during the observational period of three months.

#### 3.3.2. Qualitative Measurement

Overall, no seizures associated with hypoglycemia or ketone concentrations outside the therapeutic range were registered during the observational period. The mother reported improved cognition related to concentration and memory. No differences were observed regarding language, motor performance, social behavior, emotional stability, and sleep quality.

### 3.4. Comparison of Hypoglycemia Rate of All Three Cases

**Figure 4:**
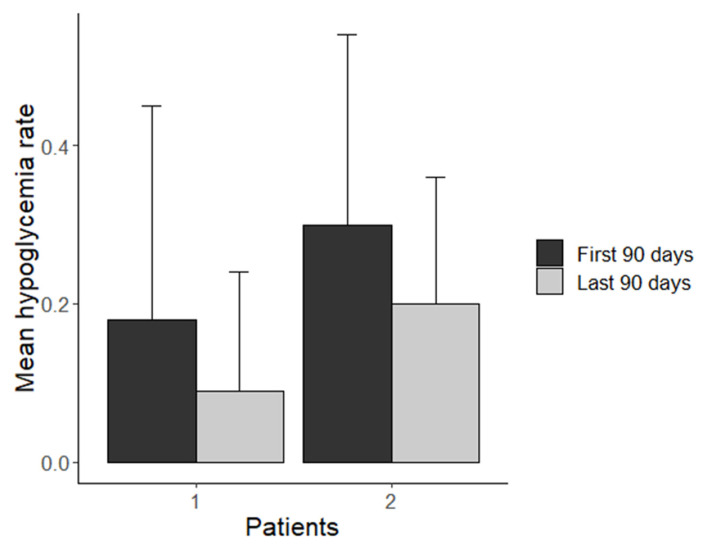
Mean hypoglycemia rate per day in the first three months and last three months of KD for patient 1 and 2.

**Figure 5:**
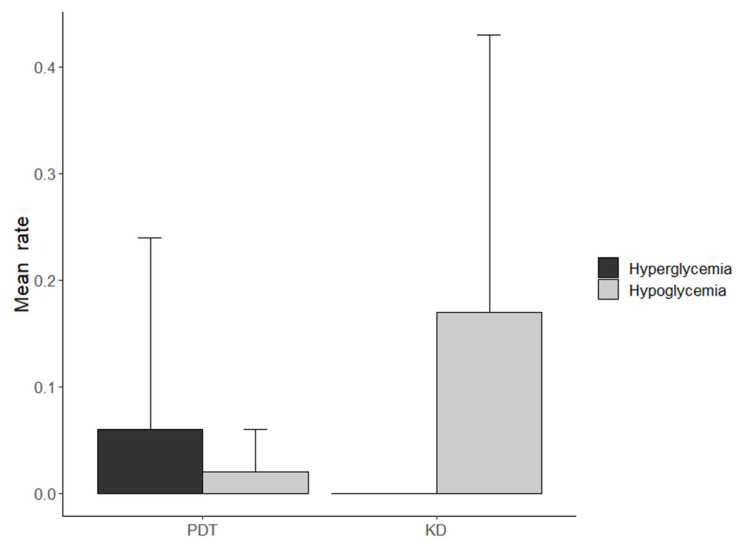
Mean hypo- and hyperglycemia rate per day in the first 30 days of PDT and KD for patient 3.

The comparison between the mean hypoglycemia rate per day of the first three months and the last three months of the KD in patient 1 and 2 are presented in Figure 4. Patient 3 switched from one-month PDT to the KD. The glucose concentration was observed for one month during PDT and three months during the KD. The 30-day period of PDT was compared to the first 30 days of the KD (Figure 5).

## 4. Discussion

In this case series, the glucose concentrations of three pediatric patients were observed during the KD and PDT as treatment options for drug resistant epilepsies in childhood. The most important finding is that hypoglycemia, as a well-known adverse effect of the KD with potential long-term harmful consequences, is especially common in the beginning of the KD and declines significantly thereafter.

Our results are, therefore, in line with our previous findings in a pediatric patient with Dravet Syndrome using an FGM system over an observational period of six months, where a decline in the hypoglycemia rate three months after the beginning of the KD was shown [19]. In addition to emesis and food refusal, hypoglycemia, as an adverse side effect of the KD, was frequently observed in younger children [11]. Especially in the early years, glycemic extremes might lead to alterations in brain structure and functions [12]. Severe hypoglycemia seems to have a negative impact on overall cognitive functioning and especially on attention and memory performance [30,31]. Additionally, chronic hypoglycemia, often observed in children with type 1 diabetes mellitus, was linked to lower cognitive outcomes in pediatric patients [12]. Moreover, the occurrence of hypoglycemic seizures had a long-term negative effect on various cognitive functions, such as perceptual, motor, memory, or attention performance [13,32].

Due to the high effort of regular blood measurements, glucose concentrations are often not controlled during the KD in clinical practice. The FGM system offers a permanent measurement of glucose concentrations and can be used for 14 days without calibration. Our two patients that were observed over six months, showed a significant decline in the hypoglycemia rate after three months. This is also in line with previous findings describing hypoglycemia, particularly during diet initiation [11]. Our third patient, who switched from PDT to the KD, had a similar hypoglycemia rate in her three-month observational period compared to the other cases. The rate of significant hypoglycemia (serum glucose level 54 mg/dL and lower) tended to decline in patient 1 and 2 but this was not statistically significant. Patient 3 showed only one significant hypoglycemic event. Furthermore, no significant changes appeared in patient 3, in switching the treatments from PDT to the KD. The hyperglycemic rate was low during PDT with no further hyperglycemic events during the KD. Hypoglycemic events were low during PDT and increased after the beginning of the KD. Interestingly, the hypoglycemia rate in patient 3 was similar to the rate of patient 1 and 2 during the KD in the first three months.

None of the children showed seizures throughout the observational period that were linked to hypoglycemia or ketone concentrations beyond the therapeutic range. Greater ketone concentrations within the therapeutic range are essential for an adequate ketosis, which plays a major role in the pathophysiological basis of the KD. However, as we found a weak negative correlation between glucose and ketone concentration, comparable to our previous findings [19], glucose levels should be monitored especially when ketone concentrations are high.

Overall, all three patients responded well to the KD and showed an improvement of the EEG up to seizure freedom. These findings are equivalent to other studies demonstrating the positive effect of the KD in children with pharmaco-resistant epilepsy [3,4,33,34]. An improvement of cognition especially in concentration and memory performance, was reported in all three cases. In two patients, motor performance, emotional stability, and sleep quality were also enhanced during the KD.

Patient 3 of our case series received PDT for one month before switching to the KD for three months. Hyperglycemia can occur as a side effect during PDT, and chronic hyperglycemia in particular may have harmful effects on brain development and cognition [22,23,25,26]. In our patient, seven hyperglycemic events were registered in total over the observational period of one month. Interestingly, the hypoglycemic events were only registered on the six days when dexamethasone was introduced. Therefore, no chronic hyperglycemia could be observed, and the registered hyperglycemic events seem to be only a short-term steroid effect on the days it was administered.

Our results showed that hypoglycemia is an adverse effect especially when starting the KD. This is in line with previous studies [19,20]. However, clinical studies reported a good tolerance of induced hypoglycemia [35] and the overall hypoglycemia rates per day of all patients were quite low. Provided that the glucose concentration is observed continuously, especially at the beginning, the KD seems to be a safe and effective treatment. During PDT in patient 3, only a few hyperglycemic events occurred, and solely on the days of dexamethasone application. Using FGM or continuous glucose measurement (CGM) systems, might be practical tools to monitor glucose and ketone concentrations when children start a KD or receive PDT, to register trends (increasing or declining concentrations).

The main limitation of this study is the low number of patients. On the one hand, the three cases cannot be compared regarding sex, age, or type of epilepsy. On the other hand, similar trends in glucose concentrations were evident in all patients, pointing to the transferability of the results to different types of drug resistant epilepsies.

## 5. Conclusions

The KD is an option in the treatment of pharmaco-resistant epilepsy in children. Hypoglycemic events occurred particularly at the beginning of the KD and declined thereafter; hyperglycemic events during PDT were rare in our case. To avoid long-term negative impacts of acute and chronic hypoglycemia, glucose concentration should be monitored regularly. The FGM or CGM systems might be a practical and easy tool for children with epilepsy who are on the KD or are receiving PDT.

## Data Availability

The data presented in this study are available on request from the corresponding author.
